# *Bacillus vallismortis* acts against ginseng root rot by modifying the composition and microecological functions of ginseng root endophytes

**DOI:** 10.3389/fmicb.2025.1561057

**Published:** 2025-04-07

**Authors:** Yang Hu, Liu-yang Yang, Meng-yuan Lei, Yi-xin Yang, Zhuo Sun, Wan Wang, Zhong-ming Han, Lin Cheng, Ze-liang Lv, Mei Han, Li-min Yang

**Affiliations:** ^1^Cultivation Base of State Key Laboratory for Ecological Restoration and Ecosystem Management of Jilin Province, College of Chinese Medicinal Materials, Jilin Agricultural University, Changchun, China; ^2^Changchun Medical College, Changchun, China

**Keywords:** ginseng, root rot, *Bacillus vallismortis*, *Bacillus velezensis*, biocontrol strain, endophytic flora, community structure

## Abstract

**Introduction:**

The endophytic microbiome serves a crucial function as a secondary line of defense against pathogen invasion in plants. This study aimed to clarify the mechanism of action of the ginseng plant growth-promoting rhizobacteria (PGPR) *Bacillus vallismortis* SZ-4 synergizing with endophytic microorganisms in the prevention and control of root rot.

**Methods:**

Ginseng root samples from a susceptible group (CK) with a disease level of 0–2 and a biocontrol group (BIO) treated with strain SZ-4 were collected. We employed high-throughput sequencing to examine the microbial community structure of ginseng roots at different disease levels, explore beneficial endophytic bacteria, and evaluate the efficacy of strain SZ-4 in mitigating root rot through synergistic interactions with ginseng endophytic flora.

**Results:**

The application of the PGPR *B. vallismortis* SZ-4 biocontrol fungicide has been found to help ginseng resist *Fusarium solani* by modulating the richness and structure of endophytic microbial populations. The endophytic bacteria HY-43 and HY-46 isolated from ginseng roots treated with *B. vallismortis* SZ-4 were identified as *Bacillus velezensis* based on morphological, physiological, and biochemical characteristics, as well as 16S rDNA and *gyr*B sequencing analyses. The endophytic bacteria HY-43 and HY-46 were combined with strain SZ-4 to generate the bacterial consortia CS4-43 and CS4-46, respectively. Both CS4-43 and CS4-46 significantly enhanced the inhibitory effects of the single strain SZ-4, as well as HY-43 and HY-46, against ginseng root rot, while also promoting plant growth.

**Discussion:**

These findings offers a theoretical foundation for studying the microecological prevention and control of ginseng diseases as well as new insights for conducting research on the efficient and precise management of plant diseases.

## 1 Introduction

*Panax ginseng*, a perennial herb with a history of use spanning over 2,000 years in China, is revered as the “king of herbs” due to its significant medicinal and economic value (Jae et al., 2012). It exhibits a wide range of pharmacological properties, including anticancer (Zhao et al., 2022), anti-aging (de Oliveira Zanuso et al., 2022), cardioprotective, and immunomodulatory effects (Liu et al., 2020). However, the unique growth characteristics of ginseng render it highly susceptible to diseases throughout its growth cycle. Infections caused by pathogenic microorganisms severely impact ginseng productivity and quality, resulting in annual economic losses exceeding 50% (Kim et al., 2012).

Ginseng root rot, caused by *Fusarium solani*, is ginseng’s most lethal and widespread soil-borne disease. It is highly infectious, has a high morbidity rate, is challenging to manage, and is often referred to as “plant cancer”. Currently, the main method of controlling soil-borne diseases in ginseng involves the application of chemical pesticides. However, excessive use of these chemicals can disrupt the soil nutrient balance, leading to ecological pollution and other related problems (Peng et al., 2005). Therefore, finding a long-term, safe, and effective disease control strategy is imperative. The use of environmentally beneficial microorganisms for the biological control of soil-borne diseases has many advantages such as safety and sustainability (Song et al., 2014). This approach is currently being researched and implemented to manage diseases affecting a wide variety of crops, including field (Cui et al., 2021) and facility-cultivated crops (Lv et al., 2024). In addition, studies on the use of *Bacillus* spp. bacteria and *Xylomycetes* spp. fungi for the prevention and control of fungal diseases have been partially reported in relation to medicinal plants such as ginseng (Wang et al., 2023).

Plant growth-promoting rhizobacteria (PGPR) are a significant group of bacterial microorganisms that include *Pseudomonas*, *Bacillus*, *Serratia*, *Rhizobium*, and many other species and genera that have been shown to have disease-promoting potential, with *Pseudomonas* being the most common and *Bacillus* being the second most common (Compant et al., 2009). PGPR inhibit harmful pathogenic microorganisms and non-parasitic inter-root microorganisms in the soil, produce plant growth-promoting metabolites, and positively regulate plant uptake and utilization of mineral nutrients. Furthermore, PGPR can enhance plant resistance to disease infestations through various biocontrol mechanisms, including competition for essential resources and favorable ecological niches, production of bacteriostatic compounds, induction of systemic resistance in plants, and regulation of microbial communities (Zhang et al., 2018). Among these, the exogenous modulation of soil microbial communities during plant cultivation is beneficial for controlling plant diseases, and related research has garnered significant attention (Hu et al., 2016). In addition, the endophytic microbiome of plants responds to adverse factors, such as disease infestations (Wu et al., 2022).

The endogenous microbiome is essential for plant growth and development and serves as a secondary line of defense against pathogen invasion (Carrión et al., 2019). When plant pathogens proliferate within a host plant, the diversity of endophytic microbes is disrupted, leading to a pathological imbalance in the endophytic microbiome that exacerbates plant diseases (Busby et al., 2016). The possibility of establishing a robust microbial barrier against pathogen infestation by effectively controlling the diversity of endophytic bacteria in plants has prompted much reflection. It has been proposed that inducing the reorganization of the plant microbiome or adjusting the relative abundance of microorganisms through the addition of plant probiotics or biocontrol agents can enhance the ability of plants to defend themselves against pathogens (Hu et al., 2016; J et al., 2019; Xiao et al., 2022); however, this area of research remains in its infancy. *Bacillus* is one of the important PGPR members (Gowtham et al., 2018). It is recognized as a biocontrol agent against plant pathogens and plays a crucial role in preventing their growth and spread (Luo et al., 2022; Wang J. et al., 2022; Wang S. Y. et al., 2022; Xu et al., 2023).

Consequently, in the present investigation, we utilized PGPR *Bacillus vallismortis* SZ-4 as the experimental subject, a strain previously demonstrated by our research group to exhibit significant antifungal activity against pathogenic fungi affecting *Panax ginseng* (Sun, 2014). This study aimed to investigate the mechanisms by which PGPR influence the ginseng endophytic flora in the prevention and control of ginseng root rot disease caused by the fungus *F. solani*. The culturable dominant bacteria were isolated and purified from ginseng roots in the presence of PGPR and *F. solani*, and endophytic bacterial strains that can synergistically inhibit *F. solani* together with *B. vallismortis* SZ-4 were screened using the co-culture plate standoff test. An external pot test was conducted to confirm the effectiveness of the bacterial consortia in preventing and controlling ginseng root rot. This work offers a theoretical foundation for studying the microecological prevention and control of ginseng diseases as well as new insights for conducting research on the efficient and precise management of plant diseases.

## 2 Materials and methods

### 2.1 Microbial and plant material

Our group isolated *B. vallismortis* SZ-4 from the rhizosphere soil of healthy ginseng plants (Sun, 2014). It is currently being stored at the Strain Collection Management Center of the China Institute of Microbiology (CGMCC No. 8273). The strains were cultured in a nutrient agar (NA) medium at 32°C. The formulation of the NA medium is provided in Supplementary Table S1.

Ginseng pathogenic fungus *F. solani*. Was provided by the Department of Plant Pathology, College of Agriculture, Jilin Agricultural University, Jilin, China, and kept at 4°C for storage. The strains were cultured on potato dextrose agar (PDA) at a temperature of 25°C. The formulation of the PDA medium is provided in Supplementary Table S1.

The ginseng plants used in this study were three-year-old plants of the “Fuxing No. 1” cultivar., purchased in April 2023 from the Fusong Ginseng Industry Technology Research and Demonstration Base in Fusong County, Jilin Province, China (42°07′33.80″N, 127°11′25″E; elevation: 532 m). The plants were provided by Jilin Ginseng King Plant Protection Technology Co., Ltd.

### 2.2 Microbial suspension preparation

#### 2.2.1 Preparation of *Fusarium solani* conidial suspension

Activated *F. solani* was inoculated onto the center of a plate filled with PDA medium and incubated at 25°C in an artificial climate chamber for 14 days. Under sterile conditions, conidia were scraped from the surface of the medium using a sterile glass slide and collected into distilled water. The suspension was then agitated with sterile glass beads for 20 min and filtered through three layers of sterile gauze. A conidial suspension with a concentration of approximately 3 × 10^4^ conidia/mL was prepared using sterile distilled water and stored at 4°C for subsequent use.

#### 2.2.2 Preparation of biocontrol bacterial fermentation broth

The preserved strains SZ-4, HY-43, and HY-46 were individually inoculated into nutrient broth (NB) medium (50 mL of medium in 100 mL vessels) and activated by shaking at 180 rpm and 32°C for 12 h. The formulation of the NB medium is provided in Supplementary Table S1. The activated cultures were adjusted to an OD600 of approximately 1.0, and 100 μL of each culture was inoculated into fresh NB medium, followed by incubation at 32°C and 180 rpm for 12 h. For the preparation of the single-agent SZ-4 fermentation broth, 1% of the SZ-4 NB bacterial culture was inoculated into beef peptone yeast extract (BPY) medium (50 mL of medium in 100 mL vessels) and incubated at 32°C and 180 rpm for 24 h. The formulation of the BPY medium is provided in Supplementary Table S1. The resulting BPY fermentation broth was adjusted to a bacterial concentration of 10^8^ CFU/mL and stored at 4°C for further use. For the preparation of the two bacterial consortia fermentation broths, 1% of the HY-43 and HY-46 NB bacterial cultures were each mixed with 1% of the SZ-4 NB bacterial culture at a 1:1 ratio and inoculated into BPY fermentation medium (50 mL of medium in 100 mL vessels). The bacterial consortia were incubated at 32°C and 180 rpm for 24 h. The resulting BPY fermentation broths were adjusted to a bacterial concentration of 10^8^ CFU/mL and stored at 4°C for further use.

#### 2.2.3 Preparation of pesticide suspensions

The carbendazim and *Bacillus subtilis* lyophilised culture were dissolved to a concentration of 0.2% (w/w). The *B. subtilis* was obtained from Shandong Muyushi Biotechnology Co. The carbendazim was obtained from Sichuan Runer Technology Co.

The bacterial and spore content was determined using the hemocytometer method (Xie et al., 2023).

### 2.3 Outdoor ginseng cultivation in pots

A study on the prevention of ginseng root rot disease was conducted from June to August 2023 at the Garden of Medicinal Plants, Jilin Agricultural University, located in Changchun City, Jilin Province, China (43°48′40″N, 125°25′1″E, 215 m). The black loam soil was collected from Fusong County, China (42°23′36″N, 127°49′18″E; elevation: 420 m), with a sampling depth of 10–15 cm below the soil surface. The baseline values of the soil were 22.77 g/kg of organic matter, 304.21 mg/kg of alkaline dissolved nitrogen (ADN), 9.49 mg/kg of active phosphorus (AP), 189.67 mg/kg of fast-acting potassium (FAP), pH 6.42, and an electrical conductivity (EC) of 335.62 dS/m. Ginseng nursery soil was a blend of black loam soil and vermiculite in a 2:1 ratio.

Three-year-old ginseng plants with compact growth and well-developed root systems were used in this study. The surfaces of the ginseng roots were sterilized with 20% sodium hypochlorite, rinsed with sterile water, and transplanted into polypropylene (PP) plastic pots (26 cm diameter, 18 cm depth), with three plants per pot. The pots were maintained in a plastic greenhouse that received 16 h of natural light at 30°C and 8 h of darkness at 15°C. Plants were watered on a regular basis. Once ginseng plants were established, giinseng with comparable growth were selected for subsequent testing.

### 2.4 Ginseng disease prevention trials and sample collection

The disease control experiments of strain SZ-4 against ginseng root rot were conducted in July 2023. An outdoor potting method was employed, and two treatment groups were established: a susceptible control group (CK) and a microbial biocontrol group (BIO). In the BIO group, ginseng roots were pre-inoculated with SZ-4 bacterial fermentation broth at a concentration of 10^8^ CFU/mL, and 50 mL of the broth was inoculated into each plant, while the CK group received an equal volume of water for each plant. After 7 d of stable colonization of the roots of BIO plants, ginseng in both the CK and BIO groups was exogenously infected with root rot through root wounding and perfusion. Specifically, 7 days after colonization ginseng pots were taken, and a wound approximately 2–3 mm in diameter was created about 1 cm below the base of the ginseng stem on one side. Then, 50 mL of a *F. solani* spore suspension with a concentration of 3 × 10^4^ conidia/mL was inoculated. Each treatment group consisted of 30 pots, with 3 ginseng plants per pot.

At 30 days post-inoculation with *F. solani*, the disease incidence of ginseng was assessed, and samples were collected. From each treatment group, 30 ginseng plants were randomly selected for sampling. The degree of ginseng disease was classified into four grades (Han et al., 2017), grade 0: healthy ginseng (CK0 and BIO0); grade 1: lesions covering less than 20% of the root surface area (CK1 and BIO1); grade 2: white mycelia appearing on the roots with lesions covering 21–40% of the root surface area (CK2 and BIO2); grade 3: lesions covering 41–65% of the root surface area, with severely affected appearance; grade 4: lesions covering more than 66% of the root surface area or completely rotted. The disease index was calculated, and the biocontrol efficacy was determined.


$$\begin{array}{l} \mathrm{Disease Index}=\\ \sum \left(\begin{array}{l} \mathrm{Number of plants in each disease grade}\\ \times \mathrm{Corresponding grade value} \end{array}\right)/\left(\begin{array}{l} \mathrm{Total number of plants}\\ \times \mathrm{Maximum grade value} \end{array}\right)\times 100 \end{array}$$

$$\begin{array}{l} \mathrm{Biocontrol Efficacy}\\ =\left[\left(\begin{array}{l} \mathrm{Disease index of control group}\\ –\mathrm{Disease index of treatment group} \end{array}\right)/\mathrm{Disease index of control group}\right]\times 100\% \end{array}$$

Since the disease severity in the biocontrol group was controlled within level 2, this study randomly collected ginseng plants with disease ratings of 0–2 from both treatment groups. Five ginseng root samples were collected for each disease rating, resulting in a total of 30 ginseng root samples. All samples were individually placed in zip-lock bags, transported to the laboratory on ice, and subsequently processed for further analysis.

### 2.5 Sample preparation for metagenomic analysis

The optimised method was employed to sterilise 30 whole-root ginseng samples. Specifically, the soil adhering to the surface of the roots was first shaken off, followed by repeated rinsing with sterile water to remove impurities from the root surface. The ginseng roots were then cut into small pieces of 0.5–0.8 cm, and 2–5 g were retained in a beaker. Subsequently, the root pieces were sequentially treated by soaking in 70% ethanol for 40 s and in 2.5% sodium hypochlorite for 10 min. After this, the root pieces were rinsed five times with sterile water, and any residual moisture was absorbed using sterile filter paper. The ginseng root pieces were then placed in cryotubes and frozen in liquid nitrogen for 1 h before being transferred to a − 80°C environment for storage until DNA extraction.

### 2.6 DNA extraction, PCR amplification, and Illumina NovaSeq sequencing

Total microbiome DNA was extracted from each sample using the CTAB method, and the quality of the DNA extraction was detected using agarose gel electrophoresis, while DNA was quantified using a UV spectrophotometer (wavelength 260 nm). For the V3–V4 region of 16S rDNA, we chose the following primers to amplify the sequence in three rounds (Walters et al., 2016): fM1 (5’-CCGCGTGNRBGAHGAAGGYYYT-3′)-rC5 (5’-TAATCCTGTTTGCTCCCCAC-3′), fM1 (5’-CCGCGTGNRBGAHGAAGGYYYT-3′)- V4-rC5(5’-GACTACHVGGGTWTCTAATCCTGTTTGCTC-3′) and 515F (5’-GTGYCAGCMGCCGCGGTAA-3′)-806R (5′- GGACTACNVGGGTWTCTAAT-3′). For the ITS, two rounds of sequence amplification were performed using the following primers (Zhu et al., 2023): ITS1F (5’-CTTGGTCATTTAGAGGAAGTAA-3′)-ITS4 (5’-TCCTCCGCTTATTGATATATGC-3′) and fITS7 (5′- GTGARTCATCGAATCTTTG-3′) - ITS4 (5’-TCCTCCGCTTATTGATATGC-3′). PCR amplification was performed using a 50 μL PCR amplification system: a 25 μL reaction system containing 25 ng of template DNA, 12.5 μL of PCR premix, 2.5 μL of each primer, and a regulated volume of PCR grade water. PCR amplification was performed using Taq standard kits purchased from Vazyme Biotech Co. The specific conditions for the endophytic fungal and endophytic bacterial PCR are described in the Supplementary Tables S2–S6. PCR products were confirmed using 2% agarose gel electrophoresis. Ultrapure water was used throughout the DNA extraction process to ensure the accuracy of the PCR results, and the PCR products were purified using AMPure XT beads (Beckman Coulter Genomics, Danvers, MA, United States) and quantified using Qubit (Invitrogen, Waltham, MA, United States). Finally, 30 samples were sequenced on an Illumina NovaSeq platform (Illumina, San Diego, CA, United States) according to the manufacturer’s recommendations (LC-Bio, Hangzhou, China provided sequencing services).

### 2.7 Microbial diversity data analysis and statistical analysis

Based on amplicon sequence variant (ASV) feature sequences and abundance tables, we performed alpha and beta diversity analyses. We selected three indices to determine the microbial alpha diversity: the number of observed operational taxonomic units (OTUs), the Chao1 index, and the Shannon index. Differences among groups were analyzed using Kruskal-Wallis tests. Beta diversity was evaluated through non-metric multidimensional scaling (NMDS) based on the Bray-Curtis distance to visualise relationships between and within communities. Additionally, the ANOSIM function was applied to assess differences in microbial communities across treatments. Both alpha and beta diversity indices were computed using QIIME2. The Wilcoxon rank-sum test was employed to identify species with statistically significant differential abundance, and bar plots were generated to analyse inter-group species abundance differences, specifically comparing the control group (CK) and the biocontrol group (BIO). Functional annotation was conducted using the Kyoto Encyclopedia of Genes and Genomes (KEGG) database, based on PICRUSt2 functional predictions (Kanehisa and Goto, 2000; Douglas et al., 2020). Spearman’s correlation coefficients were calculated to examine relationships between the relative abundances of endophytic fungi and bacteria across different disease-level biocontrol groups (BIO), with results visualised as clustered heatmaps. Statistical analyses of the ginseng disease index, control effect index, and raw data on ginseng growth and development were performed using DPS (v9.50) and Microsoft Office 2010 Excel Standard. Data visualizations, including Venn diagrams, *α*-diversity boxplots, circlize, clustered heatmaps of relative abundances, and STAMP ANOVA plots, were generated using the Venn-diagram, BoxPlot, circos, and corrplot packages in R (version 3.4.4). All data have been uploaded to NCBI under the registration numbers PRJNA1167876 bacteria^1^ and PRJNA1167695 fungi.^2^

### 2.8 Isolation and purification of endophytes

During the sampling process in the ginseng disease prevention trial, roots with disease severity levels 1–2 were selected from the BIO group. Endophytic bacteria cultivable under the combined action of PGPR *B. vallismortis* and *F. solani* were isolated and purified. The ginseng root samples were cut into 0.2 cm × 0.2 cm long segments (slices) using the tissue isolation method in aseptic conditions after surface sterilization (Sony et al., 2016). They were placed in NA medium and incubated at 32°C for 48 h. When colony formation was visible in the medium, the colonies around the plant tissues were picked and transferred into NA for purification. The samples, hereafter referred to as “HY,” were stored at 4°C for further analysis.

### 2.9 Screening of bacterial consortia

To screen for endophytic bacteria exhibiting strong compatibility and no antagonistic effects with strain SZ-4, inter-strain compatibility assays were performed (Hu et al., 2024). Specifically, the isolated endophytic bacteria and strain SZ-4 were cross-streaked onto NA medium, with three replicates per treatment, and incubated at 30°C for 24 h. The formation of inhibition zones was observed to determine the presence or absence of antagonistic interactions.

The antifungal activity of the bacterial consortia was evaluated against *F. solani*, the causative agent of ginseng root rot, using the Kirby-Bauer (K-B) disc diffusion method (Jonesa et al., 2001). The aim was to further screen for endophytic bacteria that, when combined with *B. vallismortis* SZ-4, could exhibit synergistic inhibitory effects against *F. solani*. Specifically, a filter paper disc assay was performed by inoculating *F. solani* mycelial plugs (8 mm diameter) onto PDA plates, placing sterile filter paper discs soaked in bacterial fermentation broth at four symmetrical points, and incubating at 25°C. Inhibition zones were assessed after control plates were fully colonised. After incubation at 25°C, the radius of fungal growth was measured, and the inhibition rate was calculated.

Inhibition Rate (%) = (Control Colony Radius − Confrontation Colony Radius)/ (Control Colony Radius − 4) × 100%.

### 2.10 Morphological, physiological, biochemical, and molecular characterization of endophytic bacteria

Bacterial morphology, physiological and biochemical characteristics of strain HY-43 and HY-46 were assessed following Bergey’s Manual of Determinative Bacteriology (Vos and George, 2009). The determinative tests performed and their underlying principles include: carbon source utilization tests, which assess metabolic diversity by observing growth on media containing different carbon sources (e.g., glucose, xylose, arabinose, mannitol); protein degradation tests, which evaluate proteolytic activity through casein hydrolysis; oxidase and catalase tests were performed to detect the presence of cytochrome c oxidase and catalase enzymatic activities, respectively; the Voges-Proskauer (V-P) test, which identifies acetoin production during fermentation; the oxidative-fermentative (OF) test, which distinguishes oxidative from fermentative metabolism; citrate utilization tests, which determine the ability to use citrate as a sole carbon source; gelatin liquefaction tests, which assess proteolytic activity; starch hydrolysis tests, which detect amylase activity; the methyl red test, which identifies stable acid production during glucose metabolism; hydrogen sulfide production tests, which detect the generation of hydrogen sulfide; nitrate reduction tests, which evaluate the ability to reduce nitrate to nitrite or other products; and salt tolerance tests, which assess growth on NA medium containing 10% NaCl.

Endophytic bacteria were identified by analyzing 16S rDNA and *gyr*B gene sequences (Cui et al., 2020). The bacterial 16S rRNA gene (spanning the V1–V9 hypervariable regions, ~1,500 bp) was amplified using the universal primers 27F (5’-AGAGTTTGATCCTGGCTCAG-3′) and 1492R (5’-GGTTACCTTGTTACGACTT-3′). Additionally, a conserved region of the *gyr*B gene was amplified using the specific primers UP-1 (5’-GAAGTCATCATGACCGTTCTGCAYGCNGGNAARTTYGA-3′) and UP-2r (5’-AGCAGGGTACGGATGTGCGAGCCRTCNACRTCNGCRTCNGCRTCAT-3′). The PCR system is shown in the Supplementary Table S7. The 16S rDNA amplification conditions were as follows (Zhou et al., 2021): 94°C for 5 min followed by 94°C, 1 min; 58°C, 30 s; and 70°C, 90 s for 35 cycles, then 72°C for 10 min. The *gyr*B amplification conditions were as follows: 95°C for 4 min followed by 98°C, 10 s; 62°C, 1 min; and 72°C, 2 min for 30 cycles, then 72°C for 8 min. The amplification products were subsequently detected using 1% agarose gel electrophoresis and sent to Sangon Bioengineering Corporation (Shanghai, China) for sequencing. The two sequences were aligned using the online search engine BLAST, available at GenBank^3^ and subsequently deposited in the GenBank database to obtain accession numbers. The strain HY-43 has been assigned the accession number PQ805464, and strain HY-46 has been assigned the accession number PQ805465. Phylogenetic trees of endophytic bacteria were constructed using the neighbor-joining method in Mega X software, utilizing the 16S rDNA and *gyr*B gene sequences.

### 2.11 Determination of the biocontrol efficacy of bacterial consortia against ginseng root rot

The pot experiments to evaluate the disease biocontrol efficacy of the bacterial consortia against ginseng root rot were conducted in July 2024. A pot experiment was conducted using three-year-old ginseng plants with uniform growth vigour. The outdoor pot cultivation of ginseng followed the same method as described in section 2.3. The experiment included six treatments: (1) a negative control (CK) treated with sterile water to assess natural disease progression; (2) positive control 1 (Carbendazim), a 0.2% (w/v) carbendazim solution representing conventional chemical control; (3) positive control 2 (*B. subtilis*), a *B. subtilis* suspension as a standard biocontrol agent; (4) single-strain treatment (SZ-4), *B. vallismortis* SZ-4 (10^8^ CFU/mL); (5) bacterial consortium CS4-43 (10^8^ CFU/mL); and (6) bacterial consortium CS4-46 (10^8^ CFU/mL). Carbendazim and *B. subtilis* were selected as positive controls due to their established roles in chemical and biological disease management, respectively. The inclusion of single-strain SZ-4 alongside the consortia aimed to evaluate synergistic interactions between SZ-4 and endophytic bacteria.

After the ginseng plants were established, each treatment group was administered as follows: irrigation with sterile water, application of 0.2% carbendazim, inoculation with a *B. subtilis* suspension, and inoculation with the strain SZ-4, bacterial consortia CS4-43 and CS4-46 at a concentration of 10^8^ CFU/mL, with 50 mL applied to the root zone of each plant. Seven days later, root rot disease was exogenously induced using the wounded root irrigation method by applying 50 mL of a *F. solani* spore suspension at a concentration of 3 × 10^4^ conidia/mL, as described in Section 2.4. Each treatment consisted of 10 pots, with 3 plants per pot, totalling 30 replicates per treatment. Thirty days after inoculation with *F. solani*, the incidence of disease in ginseng was assessed and samples were collected. From each treatment group, 15 whole ginseng plants were randomly selected from the replicates, placed in zip-lock bags, and transported to the laboratory. The disease index of ginseng was recorded, and the biocontrol efficacy was calculated. The formulas for calculating the disease index and biocontrol efficacy are the same as those described in Section 2.4.

Measurements of root fresh weight, root dry weight, root length, root diameter, whole plant height, whole plant fresh weight, and whole plant dry weight were conducted to assess the efficacy of the bacterial consortium in controlling ginseng root rot and its influence on ginseng growth and development. Following the collection of fresh whole ginseng samples, surface impurities were meticulously removed using clean water, and the surface moisture was subsequently absorbed with sterile filter paper. The fresh root weight was promptly determined as the wet weight of the entire root system using an electronic balance (JJ200, Jiangsu Changshu Shuangjie Testing Instrument Factory, China) with a precision of 0.01 g after washing and drying. For the dry root weight, the root systems were subjected to drying in an oven at 65°C for 48 h until a constant weight was achieved, after which they were weighed. Root length was measured as the linear distance of the longest root in the main root, extending from the junction of the root and rhizome at the base to the root tip, using a measuring tape. Root diameter was measured at the midpoint of the cross-section of the main root, 2 cm from the root base, employing a digital vernier caliper (DHGDW15068, Delixi Electric, China) with an accuracy of 0.01 mm. Concerning the whole plant morphological parameters, the height of the whole plant was measured as the vertical distance from the longest root of the main root to the highest leaf above the ground using a measuring tape. The fresh weight and dry weight of the whole plant were determined immediately after washing and drying the entire plant for the fresh weight, and after drying at 65°C for 48 h until a constant weight was achieved for the dry weight. All parameters were measured and recorded employing standardised methodologies.

## 3 Results

### 3.1 Biocontrol efficacy of strain SZ-4 in outdoor pot experiments

To investigate the biocontrol efficacy of strain SZ-4 against ginseng root rot, this study conducted outdoor pot experiments and analyzed disease progression differences between the susceptible group (CK) and the biocontrol treatment group (BIO) at 30 days post-inoculation with the pathogen (Supplementary Figure S1). The results demonstrated that, 30 days post-inoculation with the ginseng root rot pathogen *F. solani*, the disease index of the CK group was 55, whereas the disease index of the BIO group treated with *B. vallismortis* SZ-4 was 18.33, achieving a biocontrol efficacy of 66.67% (Table 1; Supplementary Table S8). These findings demonstrate that the application of *B. vallismortis* SZ-4 provides effective control against ginseng root rot caused by the pathogen *F. solani*.

**Table 1:** Biocontrol effect of strain SZ-4 on *F. solani*.

Treatment	Disease index	Biocontrol efficiency (%)
CK	55.00 ± 2.5 a	–
BIO	18.33 ± 1.44 b	66.67

CK: susceptible control group, BIO: biopreventive group; *n* = 30. Different letters in the same column at *p* < 0.05 level by LSD test.

### 3.2 Analysis of the composition of endophytic microbial communities

**Figure 1:**
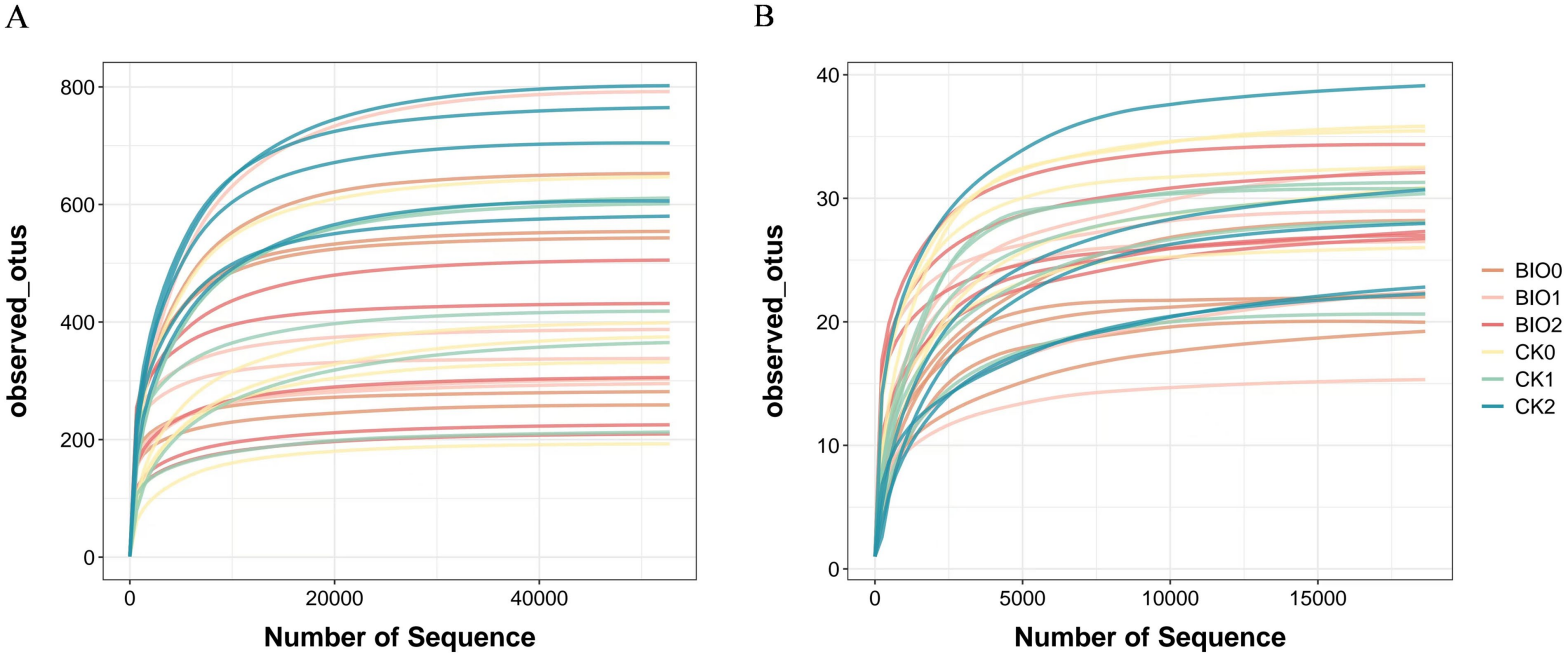
Rarefaction curves of number of OTUs recorded in ginseng roots for each treatment group in the susceptible group (CK) and biopreventive group (BIO). **(A)** Endophytic bacteria; **(B)** endophytic fungi.

The sequencing results revealed that 2,145,812 validated sequences of endophytic bacteria and 2,462,722 endophytic fungi were recovered from 30 samples, which were divided into 7,732 distinct OTUs of endophytic bacteria and 376 OTUs of endophytic fungi. Statistical analyses of the depth of sequencing of the observed OTU indices revealed that the species information was sufficiently detected in the respective samples and that the sequencing data accurately reflected the community structure and diversity of fungi and bacteria in each sample (Figure 1).

**Figure 2:**
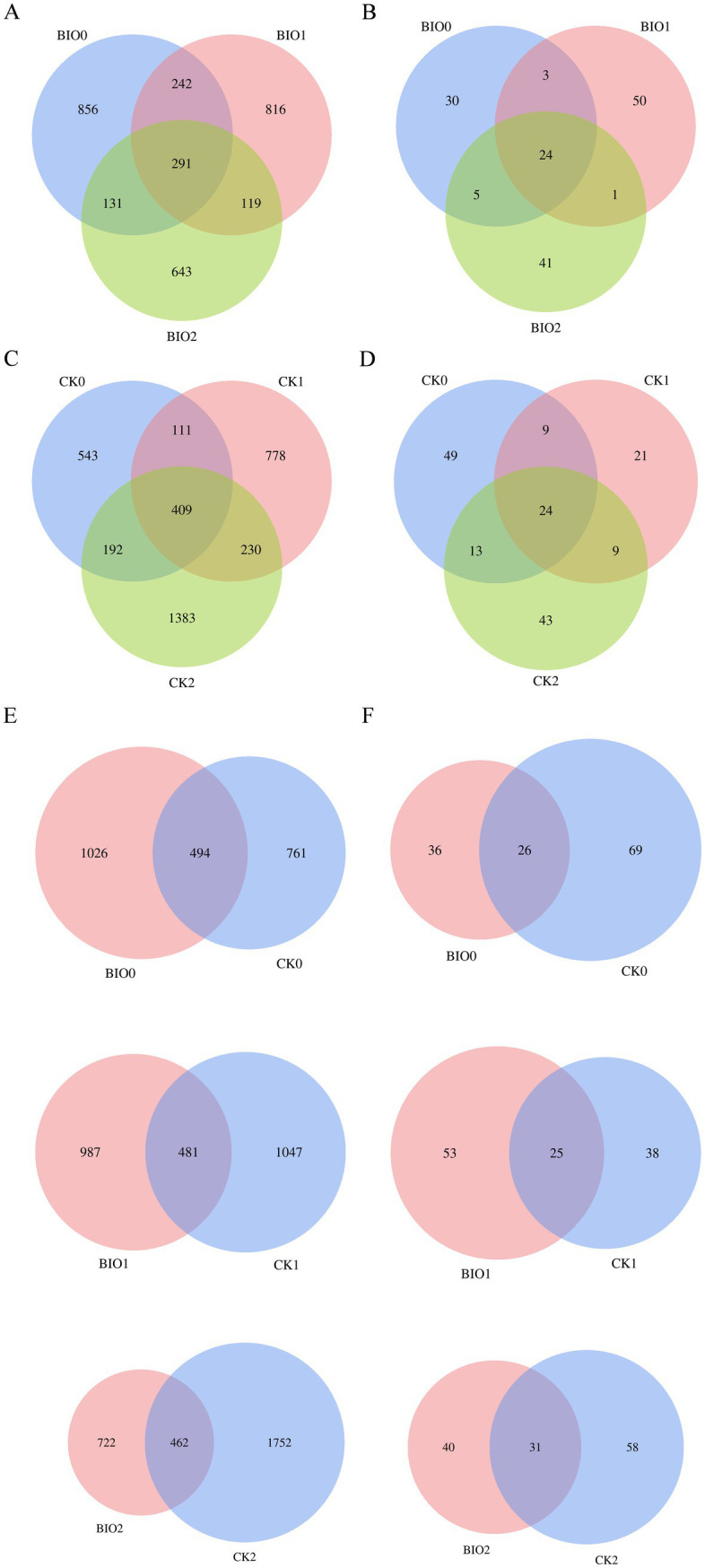
Venn diagrams showing numbers of detected OTUs at different disease levels in the susceptible (CK) and biocontrol (BIO) groups of ginseng root microorganisms. **(A,C,E)** Endophytic bacteria; **(B,D,F)** endophytic fungi.

The Venn diagram categorized the valid sequences obtained from sequencing into ASVs based on 100% similarity (Figure 2). The results indicated a significant difference in the number of ASVs between the CK and BIO groups. A total of 5,307 endophytic bacterial ASVs were identified from the ginseng samples, with the following distribution: 1,255 ASVs for CK0, 1,528 for CK1, 2,214 for CK2, 1,520 for BIO0, 1,468 for BIO1, and 1,184 for BIO2. The number of endophytic bacterial ASVs in the BIO0 group following treatment with the biocontrol agent SZ-4 was significantly higher than that observed in the CK0 group. These findings suggest that the application of SZ-4 enhances the diversity of endophytic bacterial species. As ginseng root rot progressed, the relationships between the number of endophytic bacterial ASVs in ginseng were established as follows: CK2 > CK1 > CK0, BIO0 > BIO1 > BIO2, CK1 > BIO1, and CK2 > BIO2. Additionally, the number of endophytic bacterial species in the BIO group exhibited a gradual decline correlated with the worsening of the disease, whereas an increase was observed in the CK group. Notably, ASV counts were higher in the CK1 and CK2 groups than in the BIO1 and BIO2 groups at comparable levels of disease (Figures 2A,C,E).

For endophytic fungal ASVs, 240 ASVs were identified in the ginseng samples. The distribution of ASVs was as follows: 95 for CK0, 63 for CK1, 89 for CK2, 62 for BIO0, 78 for BIO1, and 71 for BIO2. In the SZ-4-treated group, the number of ASVs in BIO0 decreased compared to CK0. Notably, the ASV counts in the diseased groups BIO1 and BIO2 were higher than those in BIO0, whereas in the control groups, the ASV counts in CK1 and CK2 were lower than those in CK0 (Figures 2B,D,F).

### 3.3 *α* and *β* diversity analysis of endophytic microbial communities

**Figure 3:**
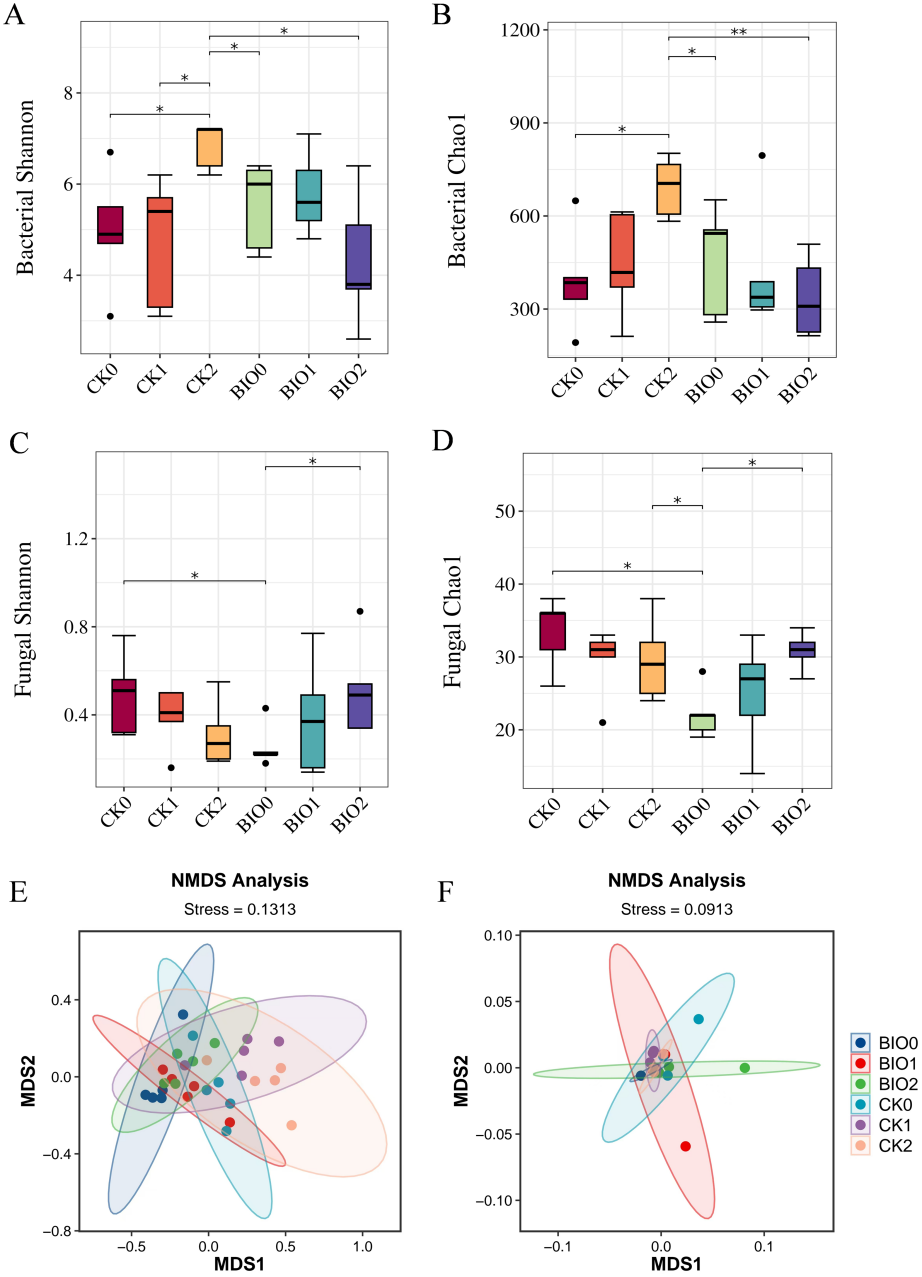
Changes in *α*-diversity of endophytic microorganisms in ginseng in the susceptible (CK) and biocontrol (BIO) groups under different levels of root rot and non-metric multidimensional scaling (NMDS) ordination plots plotted by the Bray-Curtis distance. **(A)** Shannon index of endophytic bacteria, **(B)** Chao1 index of endophytic bacteria, **(C)** Shannon index of endophytic fungi, **(D)** Chao1 index of endophytic fungi. NMDS revealed the structural differences in endophytic bacterial **(E)** and fungal **(F)** communities between the susceptible (CK) and biocontrol (BIO) groups under varying severity levels of root rot. Data are expressed as mean ± standard error (*p* < 0.05 according to Kruskal-Wallis test).

In this study, the Chao1 and Shannon indices were used to evaluate species richness and diversity, respectively. Concerning the α-diversity of endophytic bacteria, the Shannon and Chao1 indices for the BIO0 group were recorded at 5.55 and 460, respectively, whereas the CK0 group exhibited indices of 4.98 and 390, respectively. Notably, the Shannon and Chao1 indices for the BIO0 group were 11.4 and 17.9% higher, respectively, than those of the CK0 group. These findings suggest that the application of the biocontrol agent SZ-4 significantly enhanced the diversity and richness of endophytic bacteria in ginseng. With the gradual aggravation of root rot, the CK and BIO groups showed different trends with different degrees of disease severity. The Shannon index of the BIO group after disease onset showed an increasing and then decreasing trend, whereas the Chao1 index showed a decreasing trend. In contrast, the Chao1 index of the CK group showed an increasing trend, and the Shannon and Chao1 indices of the CK2 group were significantly higher than those of the CK0, CK1, and BIO2 groups (*p* < 0.05). Additionally, the Chao1 indices of BIO1 and BIO2 were lower than those of CK1 and CK2 (Figures 3A,B).

In terms of endophytic fungal α-diversity, the Shannon and Chao1 indices in the CK0 group were significantly higher than those in the BIO0 group (*p* < 0.05), indicating that treatment with the biocontrol agent SZ-4 reduced the diversity and richness of ginseng endophytic fungi. In the biocontrol group (BIO), both the Shannon and Chao1 indices showed an increasing trend with disease severity, and the indices in the BIO2 group were close to those in the CK0 group. In contrast, the Shannon and Chao1 indices in the susceptible group (CK) exhibited a decreasing trend as the disease progressed (Figures 3C,D).

All samples in the endophytic bacterial ANOSIM analysis exhibited statistically significant between-group differences (*p* < 0.05) that were greater than the within-group differences (*R* > 0). The Bray-Curtis distance-based principal coordinate analysis (PCoA) indicated no significant differences among the six sample groups. In contrast, the CK0, CK1, and CK2 samples from the susceptible control group clustered together, whereas the BIO0, BIO1, and BIO2 samples treated with SZ-4 formed a closer cluster (Figure 3E). According to the ANOSIM analysis, intergroup differences in the endophytic fungal population were greater than intragroup differences (*R* > 0). The Bray-Curtis distance-based PCoA of the endophytic fungi revealed that all six sample groups clustered together (Figure 3F).

### 3.4 Community composition and differences in endophytic microorganisms

**Figure 4:**
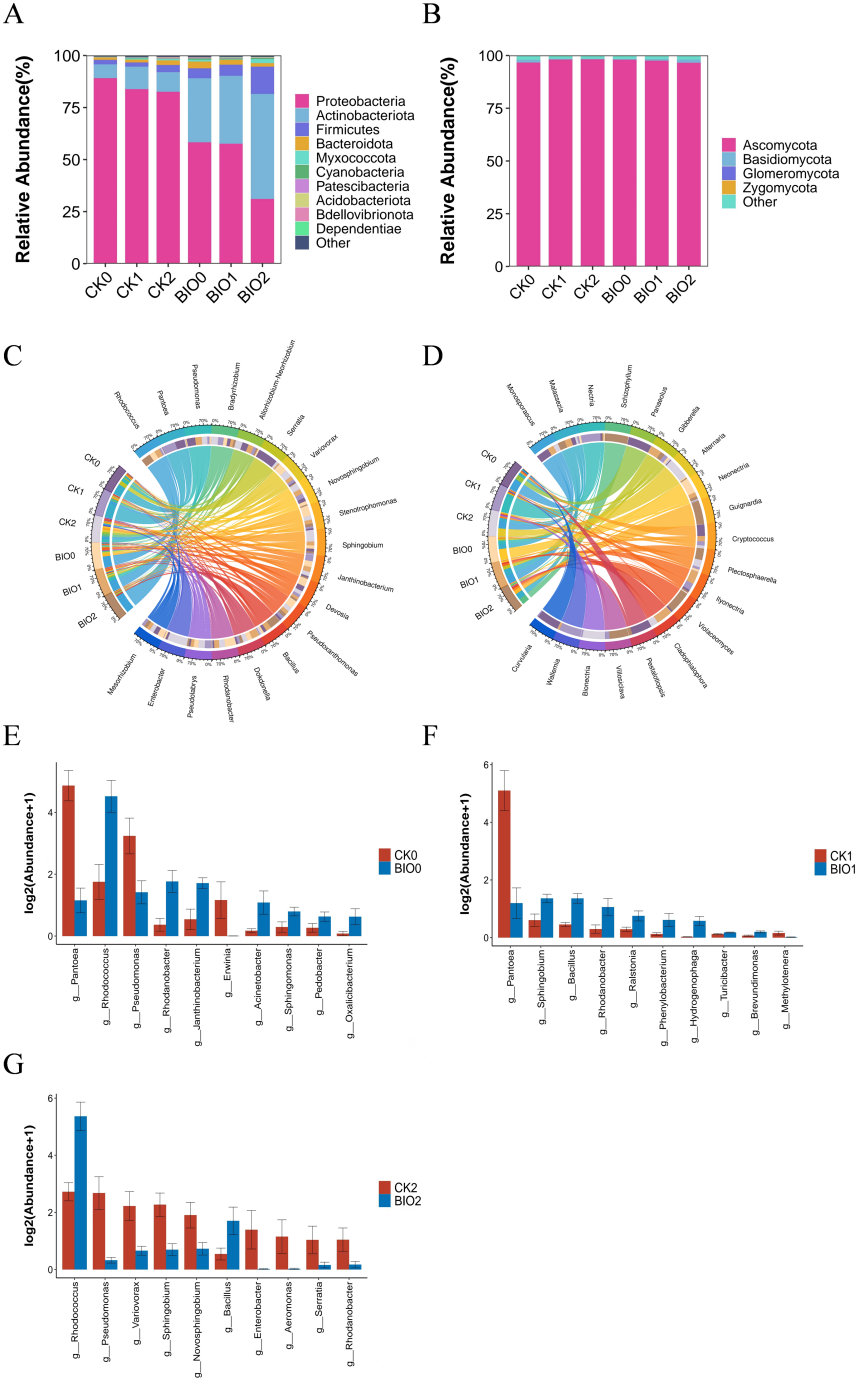
Composition and differential analysis of endophytic microbial communities in disease-susceptible (CK) and biocontrol (BIO) groups under varying disease severity levels. **(A)** Phylum-level composition and relative abundance of endophytic bacteria (top 10); **(B)** Phylum-level composition and relative abundance of endophytic fungi (top 10); **(C)** Genus-level relative proportions of endophytic bacteria (top 20); **(D)** Genus-level relative proportions of endophytic fungi (top 20); **(E–G)** Differential analysis of genus-level relative abundance (top 10) of endophytic bacteria in ginseng roots between CK and BIO groups under identical disease severity: **(E)** CK0 vs. BIO0 groups; **(F)** CK1 vs. BIO1 groups; **(G)** CK2 vs. BIO2 groups (*p* < 0.05 according to Kruskal-Wallis test).

ASV clustering was conducted on the sample sequences at a 100% similarity level. Endophytic bacteria from different samples were combined to identify 35 phyla, 102 orders, 425 families, 960 genera, and 1,596 species. The dominant phyla across all treatments were *Proteobacteria* (31–89.42%), *Actinobacteriota* (6.64–50.47%), *Firmicutes* (2–13.12%), and *Bacteroides* (1.13–3.24%). The abundance of *Proteobacteria* was reduced in all SZ-4 treated BIO groups compared to that in the CK group. Furthermore, *Proteobacteria* exhibited a decreasing trend with increasing disease severity in both CK and BIO groups. In contrast, *Actinobacteriota* and *Firmicutes* were more abundant in the BIO group, with higher levels than those observed in the CK group, and their abundance increased with disease severity (Figure 4A). Similarly, 5 phyla, 18 classes, 34 orders, 62 families, 82 genera, and 109 species of endophytic fungi were identified. At the phylum level, *Ascomycota* dominated all treatment groups (96.88–98.49%). *Ascomycota* abundance was lower in BIO0 than in CK0. As disease progressed, the abundance of *Ascomycota* in the BIO group decreased, whereas that in the CK group increased (Figure 4B).

At the genus level of root endophytic bacteria, the dominant genera in the CK and BIO groups shifted as the disease progressed, with notable differences in relative abundance. In the BIO group, *Rhodococcus* remained the most dominant genus (28–48%), while the second dominant genus varied: *Stenotrophomonas* (5.59%) in BIO0, *Pseudomonas* (8.47%) in BIO1, and notably *Bacillus* (3.33%) in BIO2. In the susceptible group (CK), the most dominant genus fluctuated due to the influence of pathogenic microorganisms. *Pantoea* was the most dominant genus in CK0 and CK1 (35–46%), while *Pseudomonas* became the most dominant genus in CK2 (7%). The second dominant genus in CK0 was *Serratia* (13.65%), whereas in CK1 and CK2, it was *Rhodococcus* (6.25 and 8.33%, respectively) (Figure 4C).

Regarding endophytic fungi, the relative abundances of *Alternaria* and *Neonectria* decreased in the BIO group as disease severity increased. *Alternaria* and *Nectria* were absent in BIO1, and *Ilyonectria* was not detected in BIO0. In the CK group, *Nectria* was absent in CK2, while *Alternaria* was absent in CK0 and CK1 but reached its highest abundance in CK2 (Figure 4D).

At the genus level, a significant difference analysis of bacteria in the roots of ginseng susceptible (CK) and biocontrol (BIO) groups at the same disease level showed that *Rhodococcus* (*p* < 0.05), *Rhodanobacter* (*p* < 0.05), *Janthinobacterium* (*p* < 0.05), and *Sphingomonas* (*p* < 0.05) were significantly higher in BIO0 than in CK0, whereas *Pantoea* (*p* < 0.01), *Pseudomonas* (*p* < 0.05), and *Erwinia* (*p* < 0.05) were significantly lower BIO0 than CK0 (Figure 4E). When comparing the relative abundances of bacteria in the roots of BIO1 and CK1, it was observed that *Sphingobium* (*p* < 0.05), *Bacillus* (*p* < 0.01), and *Rhodanobacter* (*p* < 0.05) were significantly more abundant in the BIO1 group than in the CK1 group. Conversely, *Pantoea* (*p* < 0.05) was significantly less abundant in the BIO1 group than in the CK1 group (Figure 4F). Significant differences in relative abundance of bacteria in roots between BIO2 and CK2 revealed that *Rhodococcus* (*p* < 0.05) and *Bacillus* (*p* < 0.05) were significantly higher in the BIO2 group than in CK2, while *Pseudomonas* (*p* < 0.01), *Variovorax* (*p* < 0.05), *Sphingobium* (*p* < 0.05), and *Serratia* (*p* < 0.05) were significantly lower in the BIO2 group than in CK2 (Figure 4G).

### 3.5 Fungal and bacterial correlation analysis within ginseng roots

**Figure 5:**
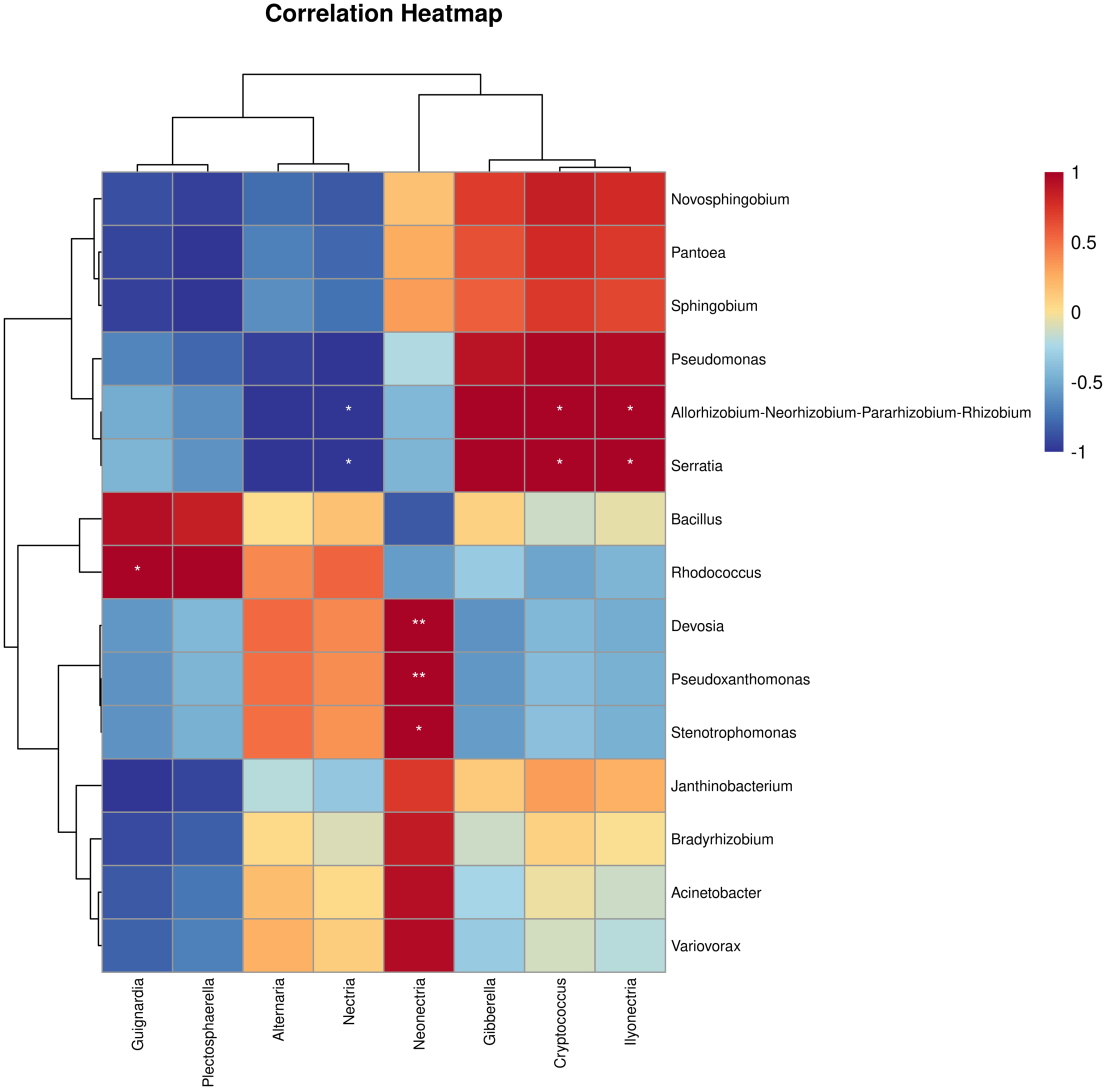
Correlation between the abundances of bacterial and fungal taxa in ginseng roots from different treatment groups in the biocontrol group (BIO) were analyzed for correlation (* and ** indicate significant correlations at *p* < 0.05 and *p* < 0.001 levels, respectively).

To gain deeper insights into the relationships among root endophytic microorganisms driven by SZ-4, Pearson correlation-based clustered heatmap analysis was performed on endophytic fungi and bacteria in the biocontrol group (BIO). The results revealed that *Serratia* exhibited a significant negative correlation with *Nectria* (*p* < 0.05) but a significant positive correlation with *Ilyonectria* (*p* < 0.05). *Bacillus* showed a negative correlation with *Neonectria*. *Rhodococcus* displayed negative correlations with both *Neonectria* and *Ilyonectria*, while *Stenotrophomonas* was negatively correlated with *Gibberella* but significantly positively correlated with *Neonectria* (*p* < 0.05). Additionally, *Pseudomonas* demonstrated negative correlations with *Alternaria* and *Nectria* (Figure 5).

### 3.6 Predictive analysis of biological functions of endophytic microorganisms

**Figure 6:**
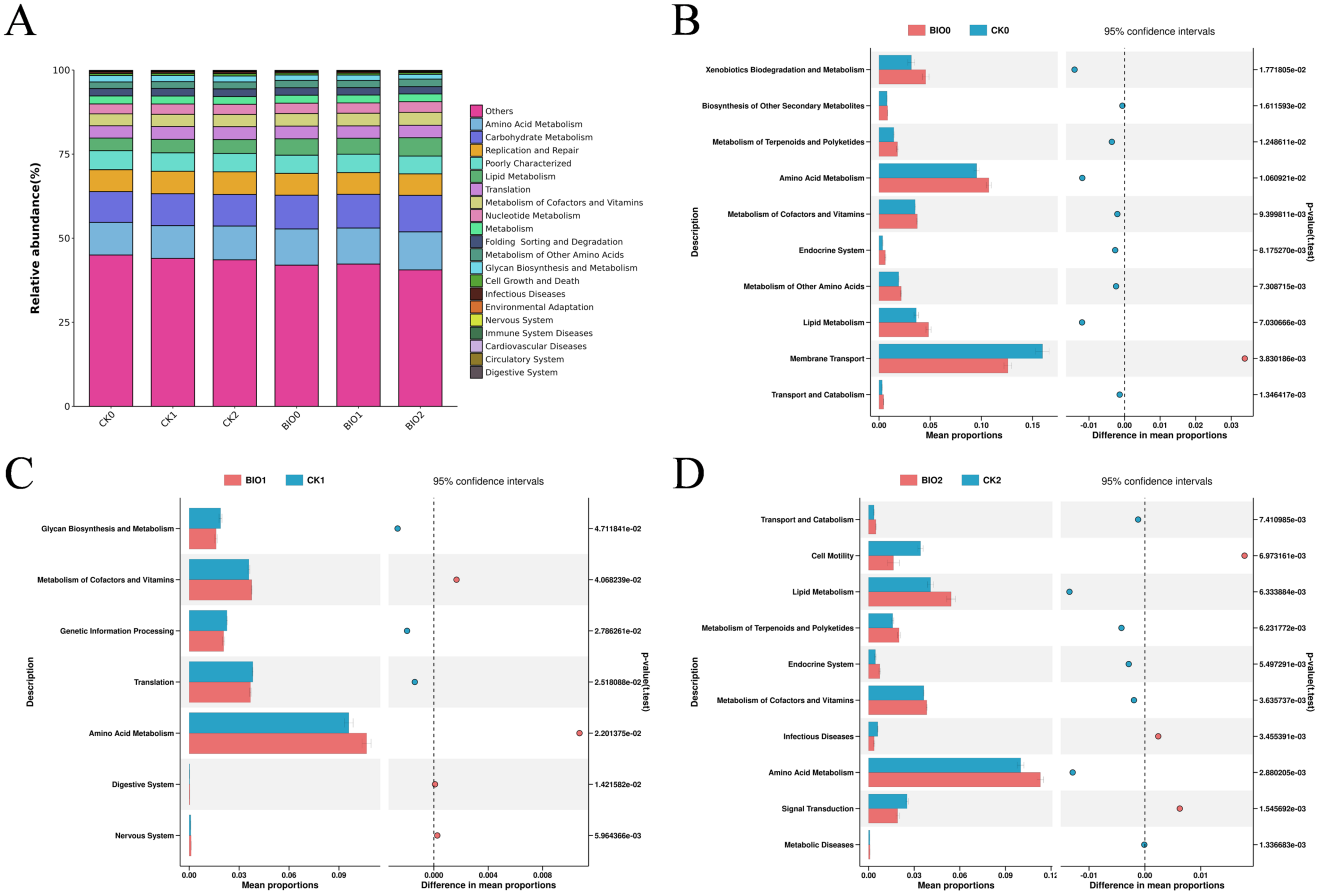
PICRUSt was used to predict bacterial ecological functions within ginseng roots. **(A)** Analysis of KEGG bacterial secondary functions for all samples; **(B)** Differences in bacterial KEGG secondary functions in roots from the CK0 and BIO0 groups. **(C)** Differences in KEGG secondary function of root bacteria in the CK1 and BIO1 groups. **(D)** Differences in KEGG secondary functions of root bacteria in CK2 and BIO2 groups. We utilized graphical chapter analysis to illustrate differences at *p* < 0.05 and Welch’s *t*-test to determine significance.

The metabolic activity of the microbial community within ginseng roots may be affected by root rot. Microbial function within ginseng roots in the biocontrol (BIO) and susceptible (CK) groups was investigated using secondary functional analysis of the KEGG pathway, utilizing PICRUSt2 to evaluate gene function in the microbial community. The findings revealed a significant degree of similarity between ecological community distributions and functional taxa across the various disease levels in the BIO and CK groups (Figure 6A). When examining the STAMP differences between the BIO and CK groups at the same disease level, the functional species enriched in BIO0 were significantly more abundant than those enriched in CK0. The BIO0 group exhibited significant enrichment in 11 functions including energy, amino acid, and carbohydrate metabolism, whereas the CK0 group was significantly enriched in six functions including membrane transport, transcription, glycan biosynthesis, and metabolism (Figure 6B). The functions enriched in the BIO1 and CK1 groups were highly variable, with four functions, including metabolism of cofactors and vitamin and amino acid metabolism, being significantly enriched in the BIO1 group, while translation, genetic information processing, and glycan biosynthesis and metabolism were significantly enriched in the CK1 group (Figure 6C). Membrane transport and 13 additional functions were significantly concentrated in the CK2 species, whereas amino acid metabolism, carbohydrate metabolism, metabolism of other amino acids, and 14 other functions were notably enriched in the BIO2 group (Figure 6D).

### 3.7 Indoor bacterial inhibition test

Using the tissue separation method, endophytic microorganisms were isolated and purified from ginseng plants co-cultured with *B. vallismortis* SZ-4 and *F. solani*. A total of 57 endophytic bacterial strains were isolated and purified, designated as HY. The isolated strains were then tested for their compatibility with SZ-4. The results showed that 49 strains exhibited compatibility with SZ-4, as no antagonistic zones were observed, indicating their potential for co-culturing with SZ-4. The 49 endophytic bacterial strains compatible with SZ-4 were further screened using the K-B disc diffusion method, resulting in the identification of 15 strains that significantly enhanced the inhibitory activity against the ginseng root rot pathogen *F. solani* when co-cultured with SZ-4 (*p* < 0.05) (Table 2). From these 15 strains, two bacterial consortia, CS4-43 and CS4-46, were selected for their pronounced antifungal activity. The inhibition rates of CS4-43 and CS4-46 against *F. solani* reached 77.8 and 78.58%, respectively, both of which were significantly higher than those of the single strains SZ-4 (75.37%), HY-43 (72.15%), and HY-46 (73.26%) (*p* < 0.05). These results demonstrate that HY-43 and HY-46 synergistically enhanced the inhibitory activity of SZ-4 against *F. solani*, confirming the synergistic effects of the co-cultured strains CS4-43 and CS4-46 in antifungal activity.

**Table 2:** Screening of bacterial consortia for inhibitory effects against *F. solani* using the Kirby-Bauer (K-B) filter paper disc method.

Bacterial consortium	*F. solani*	Bacterial consortium	*F. solani*
	Inhibition rate		Inhibition rate
SZ-4	75.37 ± 0.82 bdefgh	CS4-26	76.14 ± 0.58 abcdef
HY-43	72.15 ± 2.15 ijklmn	CS4-27	75.61 ± 1.77 bcdefg
HY-46	73.26 ± 2.01 ghijklm	CS4-28	76.50 ± 0.28 abcdef
CS4-1	70.67 ± 5.59 mno	CS4-29	69.06 ± 0.91 o
CS4-2	72.06 ± 1.25 jklmn	CS4-30	70.39 ± 1.41 mno
CS4-3	69.06 ± 0.91 o	CS4-31	69.73 ± 0.28 no
CS4-4	76.68 ± 0.21 abcdef	CS4-32	74.07 ± 0.99 efghijk
CS4-5	74.82 ± 1.22 defghij	CS4-34	70.39 ± 1.41 mno
CS4-6	77.32 ± 0.73 abcd	CS4-36	77.23 ± 1.07 abcd
CS4-7	71.39 ± 3.23 klmno	CS4-37	75.12 ± 0.30 cdefgh
CS4-8	75.28 ± 0.75 bcdefgh	CS4-38	72.68 ± 2.24 hijklm
CS4-9	70.73 ± 1.18 mno	CS4-40	75.18 ± 0.18 cdefgh
CS4-10	76.92 ± 0.40 abcde	CS4-41	69.06 ± 0.91 o
CS4-11	77.05 ± 2.32 abcd	CS4-42	75.69 ± 1.55 bcdefg
CS4-12	70.39 ± 2.35 mno	CS4-43	77.80 ± 1.84 abc
CS4-13	71.06 ± 2.24 lmno	CS4-44	77.16 ± 0.58 abcd
CS4-14	75.09 ± 1.92 cdefgh	CS4-45	74.85 ± 0.68 defghij
CS4-15	76.89 ± 3.05 abcde	CS4-46	78.58 ± 1.12 a
CS4-16	75.02 ± 1.52 cdefghi	CS4-47	71.06 ± 0.14 lmno
CS4-17	78.15 ± 1.63 ab	CS4-48	75.12 ± 3.65 cdefgh
CS4-18	70.73 ± 1.28 mno	CS4-49	76.54 ± 2.33 abcdef
CS4-19	70.73 ± 1.74 mno	CS4-52	74.78 ± 1.42 defghij
CS4-20	74.84 ± 0.46 defghij	CS4-53	73.90 ± 3.83 fghijkl
CS4-21	74.94 ± 0.13 cdefghi	CS4-54	77.41 ± 1.07 abcd
CS4-24	75.61 ± 0.75 bcdefg	CS4-55	74.65 ± 0.25 defghij
CS4-25	73.21 ± 1.53 ghijklm	CS4-57	77.13 ± 0.18 abcd

The consortium formed by strain SZ-4 and endophytic bacteria is designated as CS. For example, “CS4-46” represents a consortium composed of SZ-4 and HY-46. Data in the table are mean ± SD; *n* = 3. Different letters in the same column at *p* < 0.05 level by LSD test.

### 3.8 Identification of strains HY-43 and HY-46

**Figure 7:**
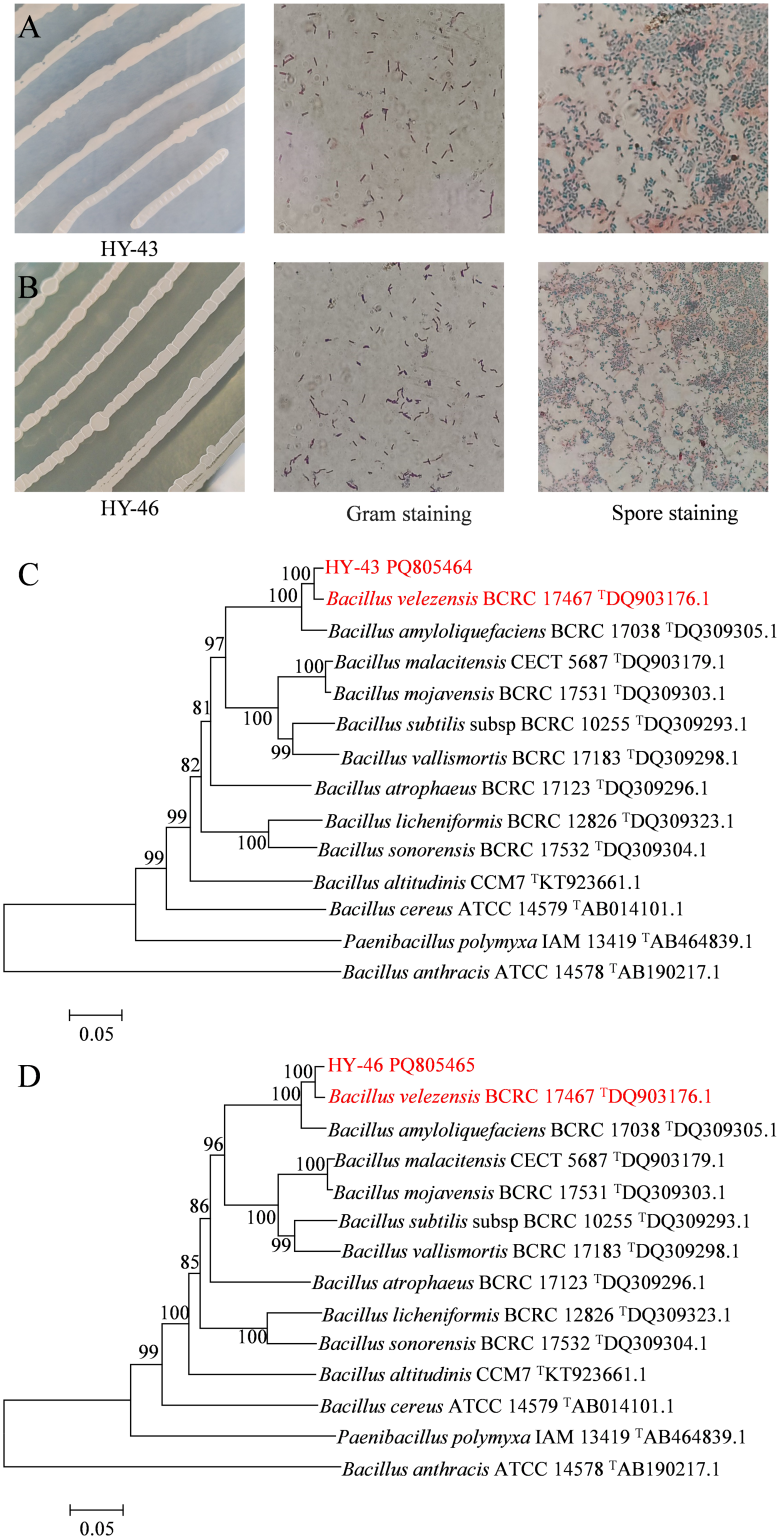
Multiple identification methods for strains HY-43 and HY-46. **(A)** Gram stain and spore stain of strain HY-43. **(B)** Gram stain and spore stain of strain HY-46. **(C)** Phylogenetic tree constructed based on partial sequences of HY-43 strain *gyr*B. **(D)** Phylogenetic tree based on partial sequence of HY-46 strain *gyr*B.

Strains HY-43 and HY-46 were inoculated onto NA solid medium and incubated at 28°C for 48 h. Both strains formed creamy white colonies that were viscous, moist, and opaque, with raised centres and smooth edges. Microscopic examination revealed rod-shaped, Gram-positive cells capable of forming endospores (Figures 7A,B). Physiological and biochemical identification results (Table 3) indicated that both HY-43 and HY-46 could utilise glucose, xylose, arabinose, and mannitol. They tested positive for casein hydrolysis, oxidase, catalase, Voges-Proskauer (V-P) test, and oxidative-fermentative (OF) test. Both strains were capable of utilizing citrate and gelatin but tested negative for starch hydrolysis, methyl red, and hydrogen sulfide production. Nitrate reduction produced a red compound, and both strains were able to grow on NA medium containing 10% NaCl. Phylogenetic analysis based on the *gyr*B gene revealed that HY-43 (GenBank accession number: PQ805464) and HY-46 (GenBank accession number: PQ805465) belong to *Bacillus velezensis* (Figures 7C,D). Therefore, based on morphological characteristics, physiological and biochemical properties, and sequencing analysis of 16S rDNA and *gyr*B, strains HY-43 and HY-46 were identified as *B. velezensis*.

**Table 3:** Physiological and biochemical characterization of strains HY-43 and HY-46.

Characteristics	HY-43	HY-46	Characteristics	HY-43	HY-46
Methyl red test	−	−	Casein Hydrolysis	+	+
D-Mannitol	+	+	Voges-Proskauer reaction	+	+
D- Xylose	+	+	Nitrate reduction	+	+
L-Arabinose	+	+	Hugh-Leifson	+	+
Glucose	+	+	Hydrogen sulfide production	−	−
Citrate utilization	+	+	Oxidase	+	+
Starch hydrolysis	−	−	Contact enzyme	+	+
Gelatin hydrolysis	+	+	Maximum salt tolerance	10%	10%

+: Positive reaction; −: Negative reaction.

### 3.9 Biocontrol efficacy of bacterial consortia CS4-43 and CS4-46 in outdoor pot experiments

To validate the synergistic effects of bacterial consortia CS4-43 and CS4-46 against ginseng root rot, this study employed outdoor pot experiments to analyse differences in disease progression among treatment groups 30 days post-inoculation with the root rot pathogen. In the water control group (CK), the most severe disease manifestation was observed, exhibiting a disease index of 80. The positive control groups treated with 0.2% carbendazim and *B. subtilis* suspension demonstrated significantly reduced disease indices of 28.33 and 30, respectively, corresponding to disease biocontrol efficacies of 64.58 and 62.5%. Application of strain SZ-4 alone achieved equivalent pathogen suppression efficacy to the chemical fungicide, registering an identical disease index of 28.33 and demonstrated a disease biocontrol efficacy of 64.58%. The bacterial consortia CS4-43 and CS4-46 demonstrated superior disease suppression, with treatment groups exhibiting respective disease indices of 23.33 and 21.67. This represents a 6.25–8.34% enhancement in biocontrol effectiveness compared to the standalone SZ-4 application, showing statistically superior efficacy to both the 0.2% carbendazim and *B. subtilis* suspension positive control groups, as well as the singular SZ-4 biocontrol agent treatment group (*p* < 0.05) (Table 4). These results suggest that the bacterial consortia CS4-43 and CS4-46 effectively controlled ginseng root rot caused by *F. solani*.

**Table 4:** Biocontrol efficiency of different treatments on *F. solani* of ginseng.

Treatment	Disease index	Biocontrol efficiency (%)
CK	80.00 ± 5.00a	–
A	28.33 ± 2.89bc	64.58 ± 3.6bc
B	30.00 ± 5.00b	62.50 ± 6.25c
C	28.33 ± 2.89bc	64.58 ± 3.61bc
D	23.33 ± 3.61 cd	70.83 ± 3.61ab
E	21.67 ± 2.89d	72.92 ± 3.61a

CK: distilled water, A: 0.2% carbendazim, B: *B. subtilis* suspension, C: strain SZ-4, D: bacterial consortium CS4-43, E: bacterial consortium CS4-46. Data in the table are mean ± SD; *n* = 15. Different letters in the same column at *p* < 0.05 level by LSD test.

### 3.10 Effect of application of bacterial consortia CS4-43 and CS4-46 on ginseng growth

**Figure 8:**
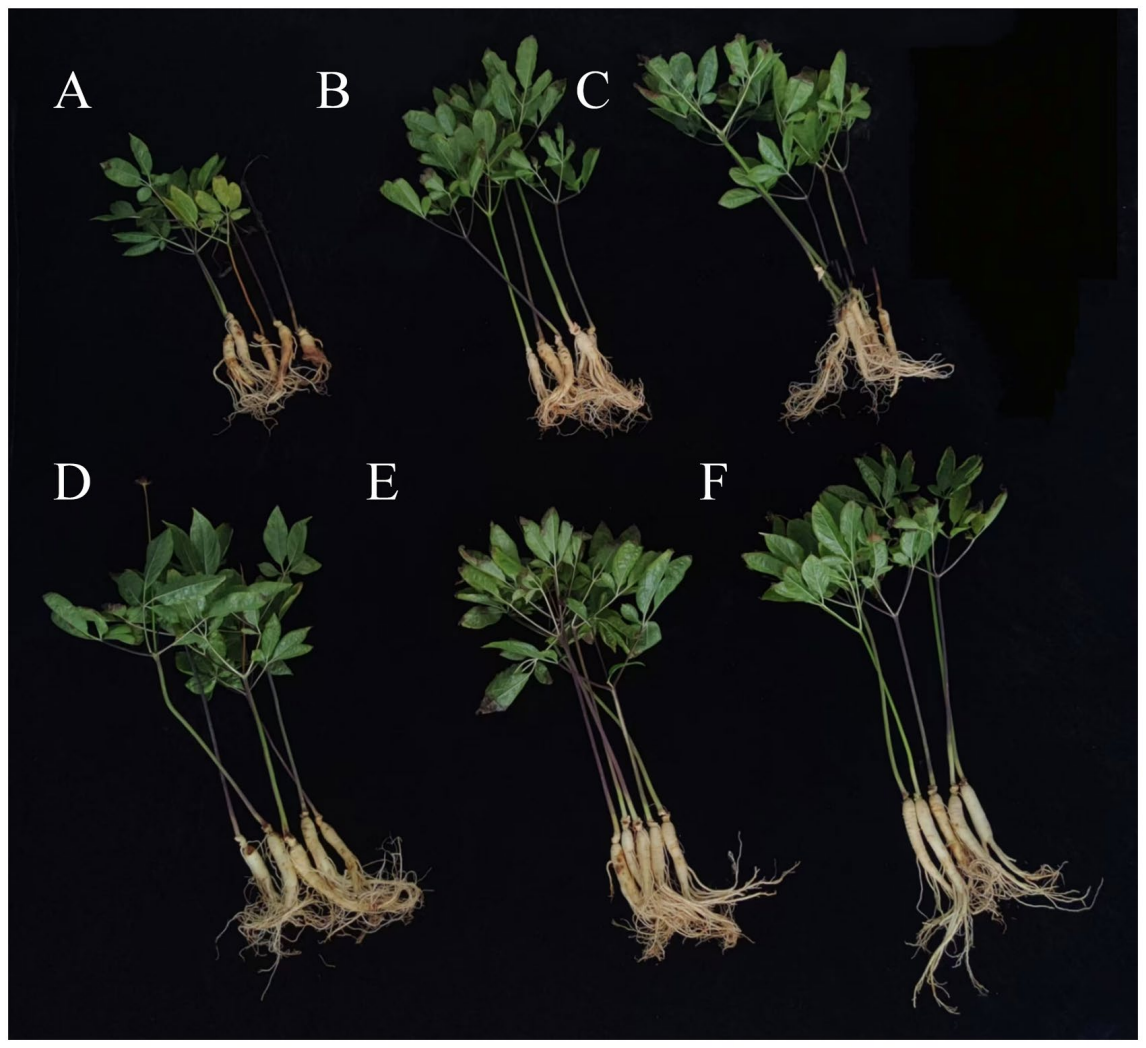
Effect of different treatments on the growth of ginseng based on its infection with root rot disease. **(A)** Distilled water, **(B)** 0.2% carbendazim, **(C)** *B. subtilis* suspension, **(D)** strain SZ-4, **(E)** bacterial consortium CS4-43, **(F)** bacterial consortium CS4-46.

Based on the effects of various treatments on the growth of ginseng infected with root rot fungus (Figure 8), the results indicated that treatment group D (bacterial consortium CS4-43) exhibited increases in several growth parameters compared with those in treatment group C (strain SZ-4). Specifically, the whole plant fresh weight, whole plant dry weight, whole plant height, root fresh weight, root dry weight, root length, and root thickness increased by 3.1, 14.01, 14.54, 10.26, 6.58, 11.73, and 7.81%, respectively. In treatment group E (bacterial consortium CS4-46), whole plant fresh weight, whole plant dry weight, whole plant height, root fresh weight, root length, and root thickness increased by 0.23, 9.43, 0.42, 6.47, 10.47, and 8.59%, respectively, compared with those in treatment group C (strain SZ-4) (Table 5). These findings suggest that treatment with the bacterial consortia CS4-43 and CS4-46 significantly improved and favoured the growth of ginseng plants.

**Table 5:** The influence of different treatments on the growth of ginseng infected with root rot.

Treatment	Whole plant fresh weight/g	Whole plant dry weight/g	Whole plant height/cm	Fresh root weight /g	Dry root weight/g	Root length/cm	Root thickness/cm
CK	9.46 ± 1.44 c	4.37 ± 0.56 c	50.57 ± 4.10 b	7.22 ± 1.50 c	1.55 ± 0.34 c	16.33 ± 0.72c	1.23 ± 0.05 c
A	14.01 ± 1.02 b	6.67 ± 0.48 b	67.33 ± 4.51 a	8.41 ± 0.55 bc	2.51 ± 0.40 b	20.55 ± 1.44b	130 ± 0.05 b
B	16.61 ± 1.40 ab	7.68 ± 0.80 ab	66.71 ± 6.96 a	10.57 ± 0.72 a	3.32 ± 0.31 a	20.32 ± 1.44b	1.45 ± 0.03 a
C	17.11 ± 3.24 ab	7.21 ± 0.64 ab	63.68 ± 4.49 a	10.04 ± 0.82 ab	3.19 ± 0.40 a	21.39 ± 0.89b	1.28 ± 0.04 bc
D	17.64 ± 2.31 a	8.22 ± 1.05 a	72.94 ± 3.74 a	11.07 ± 1.15 a	3.40 ± 0.35 a	23.9 ± 1.4a	1.38 ± 0.01a
E	17.15 ± 0.97 ab	7.89 ± 0.39 ab	63.95 ± 8.16 a	10.69 ± 0.61 a	2.96 ± 0.37 ab	23.63 ± 0.98a	1.39 ± 0.03 a

CK: distilled water, A: 0.2% carbendazim, B: *B. subtilis* suspension, C: strain SZ-4, D: bacterial consortium CS4-43, E: bacterial consortium CS4-46. Data in the table are mean ± SD; *n* = 15. Different letters in the same column at *p* < 0.05 level by LSD test.

## 4 Discussion

Microbial diversity plays a crucial role in controlling soil-borne diseases. The results of our research revealed that the number of ASVs of endophytic bacteria in the BIO0 group treated with the biocontrol agent SZ-4 was higher than that in the CK0 group, indicating that the application of SZ-4 increased the species richness of endophytic bacteria. Furthermore, the Shannon and Chao1 indices of endophytic bacteria in the BIO0 group were significantly higher than those in the CK0 group, demonstrating that the application of the biocontrol agent significantly enhanced both the richness and diversity of bacterial species. This enhanced the ability of the plants to react to the infection with *F. solani*, resulting in the severity of root rot symptoms being much less pronounced in the BIO group compared to the CK group. Exacerbation of ginseng root rot resulted in a decline in the diversity and abundance of endophytic bacteria in the biocontrol group (BIO). Conversely, there was an increase in the diversity and abundance of the endophytic fungi. Furthermore, the diversity and abundance observed in the biocontrol groups (BIO1 and BIO2) were lower than those observed in the susceptible groups (CK1 and CK2) at equivalent disease levels. It has been found that plant roots have evolved a strategy of “cry for help” to minimize the damage caused by pathogen invasion by altering or secreting various metabolites that act as signaling molecules to promote the enrichment of beneficial microorganisms when plants are infested with pathogens (Ishita et al., 2012; Neal et al., 2017; Stringlis et al., 2018; Cotton et al., 2019), and that the microorganisms within the roots of plants share similar functions. Therefore, it has been hypothesized that during the progression of disease, pathogens residing in the roots of infected plants recruit specific microbes within the plant microbiome to act as disease partners (Fulcher et al., 2024). In our research, the application of the biocontrol agent SZ-4 may elicit a “cry for help” response from the plant, which could lead to the establishment of beneficial microbial communities in ginseng roots, thereby enhancing the plant’s defensive capabilities. This interaction fosters competition among various species within ginseng roots for shared resources, including nutritional and spatial resources. Competition for ecological niches alters species richness and diversity within the root system.

Plants encounter a diverse array of biotic and abiotic factors throughout their growth cycle, with pathogen invasion representing one of the most significant biotic stressors that influence microbiome assembly (Chapelle et al., 2016; Fernández-González et al., 2020). Beneficial microbiomes can help plants overcome stress and improve their growth; therefore, it is critical for plants to effectively recruit, activate, and reorganize protective microbiomes (Rizaludin et al., 2021). In the present study, the main bacterial phyla in the ginseng roots of each treatment group were *Proteobacteria*, *Actinobacteriota*, *Firmicutes*, and *Bacteroides*, whereas the dominant endophytic fungal phylum was *Ascomycota*. *Actinobacteriota* and *Firmicutes* enhance plant resistance and development (Kumar et al., 2012). In the biocontrol group (BIO), their abundance in ginseng roots increased as the disease progressed, and the enrichment of these two microbial phyla may be a regulatory response to stress. The use of biocontrol agents during ginseng disease susceptibility may alter endophytic microbial diversity in ginseng roots. *Rhodococcus* was the dominant genus in the BIO group, whereas *Pantoea* dominated the CK group. Analysis revealed that *Rhodococcus*, *Rhodanobacter*, *Janthinobacterium*, *Sphingomonas*, and *Bacillus* were significantly enriched in the BIO group, whereas *Pantoea*, *Pseudomonas*, *Variovorax* and *Serratia* were enriched in the CK group. The diversity, abundance, and metabolic activity of microorganisms residing within plant roots are critical factors that influence plant health and management of soil-borne diseases. Populations of *Rhodanobacter* exhibit antagonistic properties against the root rot fungal pathogen *F. solani* (Huo et al., 2018) and may play a role in the nitrogen cycle (Prakash et al., 2012; Huang et al., 2021). Li et al. (2021) found that under the stress of the root rot pathogen *F. solani*, Mongolian milkvetch recruits *Stenotrophomonas*, *Achromobacter*, *Pseudomonas*, and *Flavobacterium* to the rhizosphere and rhizoplane, and a synthetic community composed of these bacteria can protect the plant by activating the synergistic effects of plant-induced systemic resistance. The beneficial bacterium *Sphingomonas* has been demonstrated to confer protection to citrus leaf rings through its competitive ability for iron uptake during infestations by the black spot pathogen (Li et al., 2022). Numerous studies have indicated that various microorganisms, including *Bacillus* spp. (Song et al., 2014; Wang et al., 2019), *Pseudomonas* spp. (Raio and Puopolo, 2021), and *Rhodococcus* spp., possess significant capabilities for disease suppression. Bacteria in the ginseng roots of the biocontrol group (BIO) treated with strain SZ-4 were associated with changes in harmful genera, and negative correlations were observed between *Bacillus* and *Neonectria*, *Stenotrophomonas* and *Gibberella*, as well as *Serratia* and *Nectria*.

Furthermore, outdoor pot experiments demonstrated that the biocontrol group (BIO) treated with *B. vallismortis* SZ-4 exhibited effective control against ginseng root rot caused by the pathogen *F. solani*, achieving a biocontrol efficacy of 66.67% and maintaining the disease severity at level 2, by the end of the 30-day-long experiment. These findings suggest that under stress from the root rot pathogen, applying the biocontrol agent SZ-4 can induce the plant to modulate the relative abundance of endophytic microorganisms, recruiting and enriching a significant number of potentially beneficial endophytic microbes. This effectively suppresses the invasion of multiple pathogens, thereby achieving the goal of controlling ginseng root rot.

PICRUSt analysis of the secondary functional layers of ginseng root bacteria revealed significant enrichment of multiple metabolic functions in the biocontrol group (BIO) during resistance to root rot, including amino acid metabolism, carbohydrate metabolism, and metabolism of cofactors and vitamins. Hildebrandt et al. demonstrated that amino acid metabolism plays a crucial role in plant disease resistance, with certain amino acids (e.g., phenylalanine and tryptophan) serving as precursors for disease resistance-related secondary metabolites, such as phenolics and phytoalexins (Hildebrandt et al., 2015). Carbohydrate metabolism provides energy and intermediate products (e.g., NADPH from the pentose phosphate pathway), which are essential for plant disease resistance (Bolouri Moghaddam and Van den Ende, 2012). Additionally, the metabolism of cofactors (e.g., NAD, FAD) and vitamins (e.g., B vitamins, vitamin C) is critical for plant defense mechanisms (Foyer and Noctor, 2011). It is hypothesized that under pathogen stress, endophytic microorganisms in the biocontrol group (BIO) enhance metabolic functions such as amino acid metabolism, carbohydrate metabolism, and metabolism of cofactors and vitamins to normal plant growth and survival, thereby maintaining active metabolic functions to improve plant stress resistance. However, due to the limitations of PICRUSt functional predictions, the genetic functions of many endophytic bacteria remain unclear. Further investigation using metagenomic sequencing and other advanced techniques is required to elucidate these mechanisms.

Currently, the use of single microbial agents for plant disease control is widespread; however, the combined application of biocontrol agents can achieve complementary advantages between strains and create synergistic effects, thereby enhancing their inherent biocontrol and growth-promoting capabilities (Huo, 2024). The findings of this study are consistent with previous research, demonstrating that the bacterial consortia CS4-43 and CS4-46 effectively promote ginseng growth and inhibit the ginseng root rot pathogen. In outdoor pot experiments, the control efficacies of bacterial consortia CS4-43 and CS4-46 reached 70.83 and 72.92%, respectively, showing significant differences compared to the carbendazim and *B. subtilis* treatment groups (*p* < 0.05). Furthermore, both consortia exhibited enhanced growth-promoting and biocontrol effects compared to the SZ-4, HY-43, or HY-46 treatments alone. These results confirm that the synthetic microbial consortia constructed by combining *B. vallismortis* SZ-4 with the endophytic bacteria with which it co-occurs in ginseng plants infected with root rot can synergistically improve the biocontrol efficacy against ginseng root rot and promote plant growth. In addition, the results of this study will contribute to the further characterization of the biological properties of endophytic bacteria with high affinity to *B. vallismortis* SZ-4 and construct synthetic microbial consortia with broad-spectrum and high-efficiency control effects against fungal diseases such as ginseng root rot. This will provide a scientific foundation for advancing research on biocontrol technologies for ginseng diseases.

## 5 Conclusion

This study revealed that the ginseng PGPR *B. vallismortis* SZ-4 significantly enhances ginseng resistance to the root rot pathogen *F. solani* by modulating the diversity and structure of the root endophytic microbial community. Furthermore, this research demonstrated that *B. vallismortis* SZ-4, through the regulation of endophytic microbial community structure and synergistic interactions with endophytic bacteria HY-43 and HY-46, significantly improves the biocontrol efficacy against ginseng root rot and promotes plant growth. These findings provide important theoretical and practical support for the microecological management of ginseng diseases through the application of synthetic microbial communities.

## Data Availability

The datasets presented in this study can be found in online repositories. The names of the repository/repositories and accession number(s) can be found in the article/Supplementary material.

## Footnotes

^1^ https://dataview.ncbi.nlm.nih.gov/object/PRJNA1167876?reviewer=gelpl7tjjacot9a9k2h3e36f59

^2^ https://dataview.ncbi.nlm.nih.gov/object/PRJNA1167695?reviewer=s1mkchug8c2qmglvaf5k9bdqpt

^3^ http://blast.ncbi.nlm.nih.gov/Blast.cgi
