# Supplementary material for: Bacillus vallismortis acts against ginseng root rot by modifying the composition and microecological functions of ginseng root endophytes
Source: Front Microbiol. 2025 Apr 7;16:1561057. doi: 10.3389/fmicb.2025.1561057 (PMC12009907; doi:10.3389/fmicb.2025.1561057)
Supplement: Supplementary file 1 [file Table_1.docx]

**Table S1**

**Media component**

| Medium | Formula | Sterilization temperature and time |
| --- | --- | --- |
| Nutrient agar medium (NA) | beef paste 3 g/L, peptone 10 g/L, sodium chloride 5 g/L, agar 20 g/L, pH 7.0-7. 2 | Sterilized at 121℃ for 30 min |
| Nutrient broth medium (NB) | beef paste 3 g/L, peptone 10 g/L, sodium chloride 5 g/L, pH 7.0-7. 2 | Sterilized at 121℃ for 30 min |
| Beef peptone yeast medium (BPY) | beef paste 5 g/L, peptone 10 g/L, yeast paste 5 g/L, glucose 5 g/L, sodium chloride 5 g/L, pH 6.8-7.0 | Sterilized at 115℃ for 20 min |
| Potato dextrose agar medium (PDA) | potato 200 g/L, dextrose 20 g/L, agar 20 g/L, pH 7.0-7.2 | Sterilized at 121℃ for 30 min |

**Table S2**

**First-round PCR reaction conditions for endophytic bacteria**

| **PCR reaction temperature** | **PCR reaction time** | **number of amplification cycles** |
| --- | --- | --- |
| 94℃ | 3 min |  |
| 94℃ | 1 min | 30 cycles |
| 70℃ | 1 min | 30 cycles |
| 54℃ | 1 min | 30 cycles |
| 72℃ | 2 min | 30 cycles |
| 72℃ | 5 min |  |
| 4℃ | ∞ |  |

**Table S3**

**Second-round PCR reaction conditions for endophytic bacteria**

| **PCR reaction temperature** | **PCR reaction time** | **number of amplification cycles** |
| --- | --- | --- |
| 98℃ | 1 min |  |
| 98℃ | 10 s |  |
| 52℃ | 30 s | 10 cycles |
| 72℃ | 30 s |  |
| 72℃ | 10 min |  |
| 4℃ | ∞ |  |

**Table S4**

**Third-round PCR reaction conditions for endophytic bacteria**

| **PCR reaction temperature** | **PCR reaction time** | **number of amplification cycles** |
| --- | --- | --- |
| 95℃ | 2 min |  |
| 95℃ | 20 s |  |
| 52℃ | 30 s | 11 cycles |
| 72℃ | 20 s |  |
| 72℃ | 10 min |  |
| 4℃ | ∞ |  |

**Table S5**

**First-round PCR reaction conditions for endophytic fungi**

| **PCR reaction temperature** | **PCR reaction time** | **number of amplification cycles** |
| --- | --- | --- |
| 94℃ | 5 min |  |
| 94℃ | 1 min |  |
| 50℃ | 50 s | 20 cycles |
| 68℃ | 1 min |  |
| 68℃ | 10 min |  |
| 4℃ | ∞ |  |

**Table S6**

**Second-round PCR reaction conditions for endophytic fungi**

| **PCR reaction temperature** | **PCR reaction time** | **number of amplification cycles** |
| --- | --- | --- |
| 94℃ | 1 min |  |
| 94℃ | 10 s |  |
| 50℃ | 30 s | 19 cycles |
| 72℃ | 45 s |  |
| 72℃ | 10 min |  |
| 4℃ | ∞ |  |

**Table S7**

**PCR reaction system**

| **Reagent** | **50 μL Reaction system composition** |
| --- | --- |
| DNA | 2 |
| dNTP(10 mmoL/L) | 2 |
| 10×Buffer | 5 |
| 27F/UP-1 | 2 |
| 1492R/UP-2r | 2 |
| Taq enzyme（2.5 U/μL） | 2 |
| ddH2O | 35 |
| Total | 50 |

**Table S8**

**Distribution of disease severity levels in the susceptible (CK) and biopreventive (BIO) Groups.**

| **Treatment** | **Disease grade (0-4 scale)** | | | | |
| --- | --- | --- | --- | --- | --- |
|  | 0 | 1 | 2 | 3 | 4 |
| **CK** | 5 | 5 | 5 | 9 | 6 |
| **BIO** | 14 | 10 | 6 |  |  |

**Figure S1**

**Comparison of sampling for potting disease control trials in susceptible (CK) and biopreventive (BIO) groups**

**
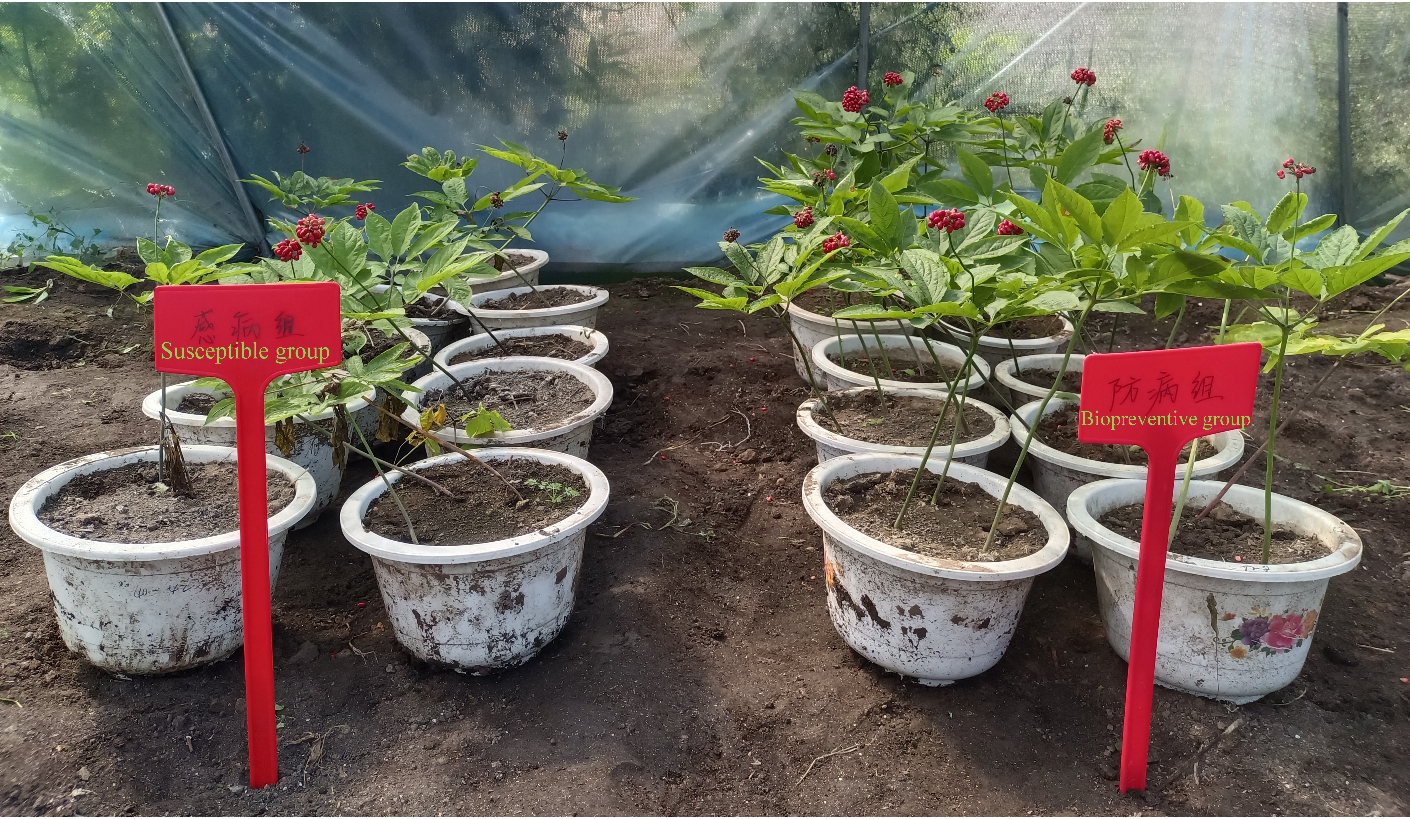
**
